# Use of Telehealth Services for Prenatal Care in Mississippi: Comparison of Pre-COVID-19 Pandemic and Pandemic Obstetric Management

**DOI:** 10.1155/2022/3535700

**Published:** 2022-01-31

**Authors:** Jennifer C. Reneker, Yunxi Zhang, Dorthy K. Young, Xiaojian Liu, Elizabeth A. Lutz

**Affiliations:** ^1^University of Mississippi Medical Center, School of Population Health, Department of Population Health Sciences, Jackson, MS, USA; ^2^University of Mississippi Medical Center, School of Population Health, Department of Data Science, Jackson, MS, USA; ^3^Mississippi State Department of Health, Offices of Health Data, Operations and Research, Jackson, MS, USA; ^4^University of Mississippi Medical Center, School of Medicine, Department of Obstetrics and Gynecology, Jackson, MS, USA

## Abstract

**Background:**

The SARS-CoV-2 (COVID-19) pandemic resulted in major shifts in service delivery for patient care not involving COVID-19 illness. The preexisting telehealth infrastructure in Mississippi allowed the state to rapidly expand the scope of telehealth programs. Little research has been done to examine the use of telehealth during the COVID-19 pandemic and its impact on the delivery of care during pregnancy and outcomes associated with pregnancy. The objectives of this study are to (1) describe prenatal care practices during the height of the first wave of the COVID-19 pandemic, compared to the immediate prepandemic time period, and (2) explore maternal and birth outcomes during these time periods.

**Methods:**

This study was conducted as a retrospective historical cohort study from medical records at one Maternal Care Level IV (Regional Perinatal Health Care Center) in Mississippi and its affiliated centers. The participant cohort was inclusive of women who received prenatal care prior to a single birth delivery between May 1, 2020, and January 31, 2021. The pandemic cohort was defined through the timeframe of the included participants' end-term prenatal care, with reference to the beginning of the COVID-19 pandemic. The prepandemic cohort received a majority of their prenatal care prior to the COVID-19 pandemic.

**Results:**

There were 1,894 women included. Among them, 620 (32.77%) completed the majority of their end-term pregnancy in the pre-COVID-19 time period and 1,272 (67.23%) completed the end-term pregnancy during the pandemic. The odds ratio for patients from the pandemic cohort of scheduling telehealth visits compared to not scheduling telehealth visits is 8.19 (95% CI: 3.98, 16.86) times the odds ratio for patients from the prepandemic cohort. The pandemic exposure as well as infant's gestational age and very low birth weight (VLBW) show significant effects on the infant's living status in the univariate logistic regression. However, after controlling for the infant's gestational age and VLBW, we did not detect a significant effect of pandemic exposure.

**Conclusion:**

This study demonstrated a very small reliance of telehealth for the medical supervision of pregnant women during the COVID-19 pandemic. This is likely because of the essential physical examinations that occur in women who are considered to be at high risk for poor maternal and birth outcomes. Additional studies on the impact of COVID-19 infection on maternal and infant outcomes are also needed as there may be important risk factors not yet identified for poor maternal or birth outcomes.

## 1. Introduction

In 2019, Mississippi had 36,634 live births with 19,884 (54.3%) among white women, 15,732 (42.9%) among black women, and 1,018 (2.8%) among women of other races [1]. The most recently published pregnancy-related mortality ratio was 33.2 deaths per 100,000 live births. This rate is 1.9 times higher than the average US ratio of 17.3 deaths per 100,000 live births for the same time period [2]. Cardiovascular disease and hypertensive conditions are the two most common causes of pregnancy-related maternal deaths in Mississippi. Similarly, the infant mortality rate for Mississippi is consistently one of the highest in the United States. In 2019, there were 322 infant deaths with an infant mortality rate of 8 deaths per 1,000 live births. The leading causes of death among infants in 2019 were birth defects, sudden unexpected infant death, and prematurity. Preterm birth, delivery prior to 37 weeks of pregnancy, is all too common in Mississippi, with about 1 in 7 neonates (14.6%) born prematurely [3]. Preterm delivery is disproportionate by race; women who report their race as black had 2,801 (17.8%) preterm births, followed by white women (2,427 (12.2%)) and other women (111 (10.9%)).

In Mississippi, there are few maternal-fetal medicine subspecialists, which results in limited access to this level of care. The University of Mississippi Medical Center (UMMC) is the state's only Regional Perinatal Health Care Center (ACOG Maternal Level of Care IV). As such, UMMC has an obstetric program designed to care for women with high-risk conditions from preconception through the postpartum period. This includes women at increased risk of fetal and maternal pregnancy complications. Care is rendered for fetal conditions, such as genetic disorders and malformations, as well as maternal conditions including hypertensive disorders of pregnancy, obesity, heart disease, diabetes, history of preterm labor, advanced maternal age, or communicable diseases. Any of these conditions can impact the health of the mother or child, and specialized maternal and fetal care is administered at UMMC. Prior to the SARS-CoV-2 (COVID-19) pandemic, these patients received care through face-to-face encounters with providers. The COVID-19 pandemic created a unique need to balance the critical need to monitor physiological aspects of prenatal care with the risk of exposure to the virus and the limited capacity within healthcare facilities overburdened with COVID-19 patient volumes.

Telehealth has long been viewed as a viable solution to the limited access to care and specialty provider shortage in rural areas of the United States. Use cases for telehealth increased as a result of the need to protect vulnerable high-risk populations from COVID-19 exposure through traditional healthcare delivery models [4, 5]. The COVID-19 pandemic resulted in major shifts in service delivery for patient care and related health programs not involving COVID-19 illness. The preexisting telehealth and information technology infrastructure in Mississippi allowed the state to rapidly expand the scope of programs that could provide services by this modality and enabled UMMC to ensure continuity of service provision. During the COVID-19 pandemic, prenatal care clinics at UMMC remained open. Providers had the option of conducting medical visits in person or through telehealth.

The Mississippi State Department of Health Perinatal High Risk Management/Infant Services System (PHRM/ISS) is a case management program intended to ensure healthy pregnancy outcomes for women with risk factors that could impact pregnancy, delivery, and newborn development and care and their infants. The voluntary program provides integrated health services through a multidisciplinary case management team. Similar to UMMC, prior to the COVID-19 pandemic, services were primarily rendered through face-to-face encounters. In response to the COVID-19 pandemic, the program incorporated telehealth services to improve access and reduce the risk of exposure. Between April 1, 2019, and March 31, 2020, only 9.0% of all case management services for pregnant and postpartum women enrolled in PHRM/ISS were completed through telehealth, whereas between April 1, 2020, and March 31, 2021, 23.6% of case management services for pregnant and postpartum women were completed via telehealth. Similarly, for infants, during these same time periods, there was a shift from 11.7% of PHRM/ISS case management visits completed via telehealth in 2019 to 57.0% of visits for infants delivered through telehealth in 2020.

Given the upshift in the use of telehealth for PHRM/ISS case management services during the COVID-19 pandemic, it is of interest to explore the use of in-person and telehealth services for the supervision of pregnant women during the same time period. Little research has been done to examine the use of telehealth during the COVID-19 pandemic and its impact on patient outcomes. The objectives of this study are to (1) describe prenatal care practices during the height of the COVID-19 pandemic, compared to the immediate prepandemic time period, and (2) explore maternal and birth outcomes during these time periods.

## 2. Methods

### 2.1. Study Design

This study was conducted as a retrospective historical cohort study using medical records from UMMC. The data obtained from UMMC were explored as individual level data.

### 2.2. IRB Determination

Institutional Review Board approval was obtained prior to study initiation from UMMC.

### 2.3. Settings

UMMC is located in Jackson, MS, and is the state's only academic medical center supporting the only OBGYN residency training program. It is classified as a Maternal Care Level IV (Regional Perinatal Health Care Center). As such, there is a significant high-risk obstetric practice. Routine prenatal care is also provided at UMMC. UMMC Grenada (Maternal Care Level I-Basic Care) is an additional site where routine prenatal care and deliveries are performed.

### 2.4. Participants

The participant cohort from UMMC was inclusive of women who were pregnant and received prenatal care at UMMC prior to a single birth delivery at UMMC between May 1, 2020, and January 31, 2021. The infants of the women included in this cohort were also included in this study. Women who delivered an infant at UMMC but received no prenatal care at UMMC and women who delivered multiples (e.g., twins) were excluded.

### 2.5. Variables

This study evaluates the impact of prenatal care management before and during the COVID-19 pandemic on participants' pregnancy outcomes. We define the pandemic cohort through the timeframe of the included participants' end-term prenatal care, with reference to the beginning of the COVID-19 pandemic. “End-term prenatal care” was defined as the prenatal care received during the last 1/3 of a woman's pregnancy. If the majority of a woman's end-term prenatal care was received prior to April 1, 2021, which marked the beginning of the COVID-19 pandemic, they were in the prepandemic cohort. If the majority of a woman's end-term prenatal care was received on or after April 1, 2021, they were in the pandemic cohort. This exposure status was the primary variable of interest.

Other variables of interest included demographic characteristics of the mother (age, race, and ethnicity), prenatal visit characteristics (number of encounters, primary service payment mechanism, and mode of visit), and health status of the mother (high-risk pregnancy, e.g., preexisting hypertension, hypertension during pregnancy, diabetes, and infectious diseases).

Outcomes for the mother included the delivery method (vaginal or cesarean section), length of stay, and development of preeclampsia or eclampsia. Outcomes for the infant included the infant's sex, the gestational age at birth, the birth weight, whether the infant was placed in the NICU, the length of stay, and the discharge status (i.e., alive or not).

### 2.6. Data Sources/Measurement

All medical data were extracted by an honest broker from medical records located in Epic at UMMC. The participant's race/ethnicity was recorded based on self-report; visit characteristics were based on encounter records, and health exposure and outcomes were based on ICD-10-CM codes and delivery and discharge records. Dates were used to calculate all ages and timeframes (e.g., length of stay).

### 2.7. Quantitative Variables

The gestational age at birth has been shown to be a significant factor in pregnancy outcomes [6]. Thus, we grouped all participants based on the infant's gestational age at delivery. If an infant was delivered before 37 gestational weeks, then it was considered preterm birth. Otherwise, infants delivered at 37 gestational weeks or more were considered term births. We evaluated the pandemic impact using all participants as well as by birth groups.

### 2.8. Statistical Methods

Descriptive statistics (i.e., mean with standard deviation (SD) for continuous variables and frequency with percentage for categorical variables) were used to summarize predictors and outcomes for all participants as well as by cohort (relative to the pandemic) and birth groups. Moreover, we examined the association between variables and pandemic exposure for all participants as well as by birth groups. We conducted Wilcoxon rank-sum tests and chi-squared tests, respectively, for continuous and categorical variables. Fisher's exact test was used if one of the expected values in categorical variables was less than five.

When a significant association was detected between pandemic exposure and pregnancy outcomes, a regression model was used to further evaluate the impact of pandemic exposure on pregnancy outcomes. The logistic regression model was performed on the status of the infant (alive/dead). First, a univariate logistic regression model of the infant's living status for each exposure and related pregnancy outcome was built. Then, pandemic exposure and other significant predictors identified in the univariate model were incorporated into the multivariate logistic regression model. To avoid multicollinearity, we used the presence/absence of a very low birth weight (yes/no) to represent the effect of the infant's birth weight in the regression model. If the infant's living status had a small count in any categorical predictors, the exact conditional analysis was used for estimation. An alpha level of 0.05 was used to determine statistical significance. All data analyses were conducted using SAS statistical software (version 9.4, SAS Institute Inc., Cary, NC).

## 3. Results

The baseline characteristics of mothers in this study are presented in Table 1. There were 1,894 women who delivered a single infant at UMMC between May 1, 2020, and January 31, 2021. Among them, 620 (32.77%) completed the majority of their end-term pregnancy between May 1, 2019, and March 31, 2020, and 1272 (67.23%) completed the end-term pregnancy between April 1, 2020, and January 31, 2021. Two mothers cannot be classified due to the missing gestational age. A total of 146 infants from the prepandemic cohort and 338 from the pandemic cohort were considered to be preterm. The relationship between preterm birth and COVID-19 pandemic exposure was not significant (*P* value = 0.157).

The average age of mothers at delivery was 27.31 years (SD = 6.05). On average, mothers who had preterm birth were 1.75 years older than those who had term births. Over 68% of mothers in this study were non-Hispanic African Americans, and 21% of mothers were non-Hispanic Caucasians. Commercial and Medicaid were the two major financial classes used by mothers in this study. Age at delivery, ethnicity and race, and payment methods were not significantly different between pre-COVID-19 pandemic and pandemic cohorts or between birth groups.

In total, 32,758 encounters occurred at UMMC, and only 226 (<1.0%) of them were scheduled for telehealth. The average number of encounters for the pandemic cohort increased by 0.69 from that in the prepandemic cohort, but this change was not statistically significant. However, the odds ratio for patients from the pandemic cohort of scheduling telehealth visits compared to not scheduling telehealth visits is 8.19 (95% CI: 3.98, 16.86) times the odds ratio for patients from the prepandemic cohort. The scheduled telehealth odds ratio is 14.12 (95% CI: 1.91, 104.58) for the preterm birth group and 7.38 (95% CI: 3.39, 16.04) for the term birth group. The average number of telehealth visits scheduled for each patient in the pandemic cohort increased to around eight times that of the patients in the prepandemic cohort (*P* value < 0.001). Moreover, the pandemic exposure had a highly significant association with the use of telehealth for both preterm birth and term birth groups.

The pandemic cohort had a higher prevalence of high-risk pregnancies, including hypertension during pregnancy, and infectious diseases, especially for the term birth group. The supervision of high-risk pregnancy was diagnosed in 39% of the prepandemic cohort and 54% of the pandemic cohort. In the term birth group, there was an 18% increase from prepandemic to pandemic cohorts who were diagnosed with high-risk pregnancy. Additionally, hypertension during pregnancy was diagnosed in 8% of the prepandemic cohort and 14% of the pandemic cohort. In the term birth group, there was a 5% increase from the prepandemic cohort to the pandemic cohort. Furthermore, the number of mothers who were diagnosed with infectious diseases also increased during the pandemic. The presence of one or more infectious diseases in pregnancy was diagnosed in 21% of the prepandemic cohort and 31% of the pandemic cohort. In the term birth group, the proportion of mothers with infectious diseases increased by over 10%.

Table 2 displays the pregnancy outcomes for mothers and infants. The mean gestational age of all the infants in this sample was 36.82 weeks and was not significantly different by exposure status. Across all infants, 7.55% of births were considered to have a VLBW. The infant's living status was the only outcome detected with a significant association with the pandemic exposure (*P*=0.034). The percentage of infants living at discharge was 96.77% for the prepandemic cohort and declined to 94.58% for the pandemic cohort.

Table 3 displays the results of logistic regression models. The pandemic exposure as well as infant's gestational age and very low birth weight (VLBW) show significant effects on the infant's delivery status in the univariate logistic regression. However, in the multivariate logistic regression model, after controlling for the infant's gestational age and VLBW, we did not detect a significant effect of pandemic exposure. Specifically, the result of the multivariate logistic model showed that, for each 1 week older in gestational age, an infant had 39% higher odds of living at the time of discharge. Also, infants with a VLBW had 75% lower odds of living at the time of discharge.

## 4. Discussion

### 4.1. Key Results

First, a strong majority of prenatal care medical visits were completed in person during both time periods. Second, the number of in-person prenatal care visits was not different between the pre- and post-COVID-19 pandemic. During the COVID-19 pandemic time period represented by these data, a majority of pregnancies were considered high risk, with a higher proportion of women having infectious diseases and hypertension. Furthermore, the prevalence of these conditions significantly increased in the pandemic time period compared to the prepandemic period. There were no significant differences in any maternal pregnancy-related outcomes between the pre-COVID-19 and COVID-19 pandemic time periods examined. There were significantly more infant deaths during the pandemic period, but when explored through multiple variable regression, these differences were not attributable to the pandemic time period.

Although there were significantly more telehealth visits for prenatal care completed during the COVID-19 pandemic time period, these visits accounted for less than 1% of all prenatal care visits. Therefore, although statistically significant, this result cannot be interpreted as a clinically meaningful result. As presented in the Introduction, during these same time periods, case management services provided through the MSDH PHRM/ISS program to pregnant women, some of which were likely in the sample for this study as well, demonstrated a large increase in the use of telehealth during the COVID-19 pandemic. While we expected a similar increase in the use of telehealth for prenatal care at UMMC, we also recognize that there are differences between the essential support services provided to pregnant women for MSDH case management and the essential examinations completed during prenatal care medical visits.

Telehealth allows providers to maintain substantially equivalent patient care for many medical specialties, including some obstetric and gynecologic services [4, 7, 8]. In light of the COVID-19 pandemic, some settings implemented a rapid increase in the proportion of prenatal care delivered through telehealth [9]. It is recognized, however, that while telehealth may be used for some prenatal care visits even in high-risk pregnancies, in-person visits are still required for fetal surveillance and interventions [10]. According to the American College of Obstetricians and Gynecologists (ACOG) practice bulletin on fetal surveillance [11], fetal testing including biophysical profile and Doppler velocimetry assessments should be completed 1-2 times per week with ultrasonography completed every 3-4 weeks to monitor for growth, beginning at 32 weeks for at-risk patients, including those with preexisting maternal conditions (i.e., diabetes mellitus). This bulletin goes on to recommend that, in women with chronic hypertension and suspected fetal growth compromise, these types of tests may begin sooner. Abnormal tests can then assist in the determination of the risk-benefit of inducing an early delivery [11].

At this time, these types of examination techniques are only possible in person; current telehealth technologies cannot assist a physician perform these types of physical examinations. Given the propensity for the women in this sample to be at high risk, including high rates of DM and preexisting hypertension, the low reliance on telehealth for prenatal care is being interpreted as a positive finding. It indicates that maternal care needs, necessitating in-person examination, were prioritized over the desire to limit in-person contact. Additionally, the lack of difference in the mean number of prenatal care visits between the women in the prepandemic and pandemic cohorts further indicates that maternal/fetal health was prioritized during the COVID-19 pandemic.

A second finding demonstrated no significant difference in maternal outcomes between the prepandemic and pandemic cohorts. This included the maternal length of stay, incidence of preeclampsia or eclampsia, and delivery method. This was another positive outcome. During the pandemic timeframe, many hospitals, including UMMC, experienced a sharp rise in the number of inpatients with complications related to COVID-19 infection. Because of limited beds and personnel resources in any hospital system, it is possible that non-COVID-19 patients, including postpartum women, may have been discharged more quickly after delivery. Our data demonstrate the opposite; the length of stay was unchanged between the prepandemic and pandemic periods.

A negative finding was the increased incidence of neonate or infant death within the pandemic cohort, compared to the pre-COVID-19 cohort, rising from 3.23% to 5.42% within the sample. When accounting for the infant's gestational age and VLBW, being in the pandemic cohort was no longer significant. While the relationship between the risk of death, VLBW, and gestational age is well understood, we cannot eliminate the possibility of COVID-19 contributing to this finding [12, 13]. Within our data, we are unable to ascertain whether preterm deliveries were spontaneous or medically indicated. A published cohort study of 50 women from UMMC with COVID-19 infection revealed a significantly higher risk of preeclampsia than women who delivered prior to the pandemic (and therefore had no COVID-19 infection) [14]. Since the pandemic cohort included in this present study included a greater prevalence of preexisting hypertension, it is plausible that some of the premature births were due to early inductions or deliveries in the best interest of the fetus and mother, which may or may not have included a history of COVID-19 infection in the mother. Unfortunately, we are not able to confirm this, as history of COVID-19 infection was not included in this dataset and is beyond the scope of this paper. Furthermore, it is also possible that unknown environmental or behavioral conditions existed because of the COVID-19 pandemic, which may have contributed to any of the baseline differences between patients in the prepandemic and pandemic time periods. These factors are of interest to note and may be explored in greater detail in the future.

Conducting a retrospective review of medical records is not without limitations. Data from medical records are not collected for the purposes of research, and as such, there are several potential risks of bias, including missing data and recording errors [15]. However, these types of studies are very beneficial to describe the impact of rapidly evolving real-world exposure and medical outcomes, such as with COVID-19. This study was conducted in a research university where all data were regularly cleaned and stored in the institutional enterprise data warehouse to support research. The data of this study were retrieved by honest brokers, using software to query medical records for data fields. None of the data were extracted manually or from free-text entries. As such, the potential for transcribing errors is limited.

## 5. Conclusion

This study demonstrated a very small reliance of telehealth for the medical supervision of pregnant women during the COVID-19 pandemic. This is likely because of the essential physical examinations that occur in women who are considered to be at high risk for poor maternal and birth outcomes. Future work could explore the feasibility of telehealth for early-stage pregnancy prenatal care (i.e., first trimester) in high-risk pregnancies and possibly also remote patient monitoring as adjuncts to in-person visits. Additional studies on the impact of COVID-19 infection on maternal and infant outcomes are also needed as there may be important risk factors not yet identified for poor maternal and infant outcomes.

## Figures and Tables

**Table 1:** Mother's baseline characteristics.

	All patients (*N* = 1894)	Prepandemic cohort		Pandemic cohort		*P* value^†^
		Preterm birth (*n* = 146)^a^	Term birth (*n* = 474)^b^	Preterm birth (*n* = 338)^c^	Term birth (*n* = 934)^d^	
Age at delivery, mean (SD), y	27.31 (6.05)	28.76 (6.38)	27.19 (5.90)	28.56 (6.23)	26.70 (5.92)	0.191
Ethnicity/race, no. (%)						0.234
Non-Hispanic/Caucasian	392 (20.97)	28 (19.44)	109 (23.19)	60 (18.07)	195 (21.13)	
Non-Hispanic/African American	1274 (68.11)	106 (73.61)	294 (62.55)	255 (76.81)	618 (66.96)	
Non-Hispanic/other race	69 (3.69)	—	24 (5.11)	—	34 (3.68)	
Hispanic	135 (7.22)	—	43 (9.15)	—	76 (8.23)	
Number of encounters, mean (SD)	17.30 (13.85)	15.69 (13.90)	17.20 (13.28)	16.38 (15.38)	17.95 (13.52)	0.259
Number of mothers scheduled for telehealth encounters, no. (%)^ac∗∗∗bd∗∗∗^	131 (6.92)	—	—	30 (8.88)	93 (9.96)	**<0.001**
Primary financial class						0.443
Commercial insurance	874 (46.15)	52 (35.62)	225 (47.47)	124 (36.69)	472 (50.54)	
Medicare	29 (1.53)	—	—	—	11 (1.18)	
Medicaid	843 (44.51)	78 (53.42)	198 (41.77)	185 (54.73)	381 (40.79)	
Others	148 (7.81)	—	—	—	70 (7.49)	
Supervision of high-risk pregnancy (O09), no. (%)^bd∗∗∗^	933 (49.26)	78 (53.42)	166 (35.02)	192 (56.80)	496 (53.10)	**<0.001**
Hypertension						
Preexisting hypertension (O10 and/or O11), no. (%)	412 (21.75)	57 (39.04)	67 (14.14)	129 (38.17)	158 (16.92)	0.205
Hypertension during pregnancy (O12 and/or O16), no. (%)^bd∗∗^	233 (12.30)	22 (15.07)	29 (6.12)	76 (22.49)	106 (11.35)	**<0.001**
Diabetes (O24), no. (%)	300 (15.84)	31 (21.23)	57 (12.03)	86 (25.44)	126 (13.49)	0.167
Infectious diseases (O98), no. (%)^bd∗∗∗^	532 (28.09)	28 (19.18)	105 (22.15)	92 (27.22)	307 (32.87)	**<0.001**

P values in the last column are for the comparisons between pre-COVID-19 pandemic (*n* = 620) and pandemic (*n* = 1272) cohorts. Bold value indicates significance at 0.05 level. The significant results of comparisons between pre-pandemic and pandemic cohorts for preterm term births are marked after the variable name in the first column (^*∗*^*P* < 0.05; ^*∗∗*^*P* < 0.01; ^*∗∗∗*^*P* < 0.01). ^a^, ^b^, ^c^, and ^d^ indicate pre-pandemic cohort with preterm birth, pre-pandemic cohort with term birth, pandemic cohort with preterm birth, and pandemic cohort with term birth, respectively. Numbers less than 10 and the smallest number in the corresponding column were not reported to avoid releasing identifiable information.

**Table 2:** Pregnancy outcomes.

	All patients (*N* = 1894)	Prepandemic cohort		Pandemic cohort		*P* value^†^
		Preterm birth (*n* = 146)^a^	Term birth (*n* = 474)^b^	Preterm birth (*n* = 338)^c^	Term birth (*n* = 934)^d^	
Mother's outcomes						
Mother's hospital length of stay, mean (SD)	3.44 (4.27)	5.25 (7.69)	2.84 (1.45)	5.30 (7.98)	2.78 (1.16)	0.864
Preeclampsia or eclampsia (O14, O15), no. (%)	301 (15.89)	52 (35.62)	45 (9.49)	111 (32.84)	93 (9.96)	0.827
Delivery method, no. (%)						0.532
Vaginal^§^	1071 (56.61)	58 (39.73)	299 (63.08)	138 (40.95)	574 (61.52)	
C-section	821 (43.39)	88 (60.27)	175 (36.92)	199 (59.05)	359 (38.48)	
Infant's outcomes						
Infant's gestational age, mean (SD), weeks	36.82 (3.57)	32.80 (4.39)	38.29 (1.03)	32.29 (4.66)	38.35 (1.06)	0.566
Infant's gender, no. (%)						0.207
Male	966 (51.03)	73 (50.00)	230 (48.63)	176 (52.07)	486 (52.03)	
Female	927 (48.97)	73 (50.00)	243 (51.37)	162 (47.93)	448 (47.97)	
Infant's hospital length of stay, mean (SD)	7.76 (19.27)	21.53 (33.20)	3.76 (7.79)	19.87 (32.59)	3.29 (7.58)	0.293
Birth weight						
Infant's admitting birth weight, mean (SD) (g)	2910 (690)	2213 (731)	3132 (474)	2146 (756)	3147 (461)	0.812
Low birth weight (less than 2500 g), no. (%)	451 (23.81)	96 (65.75)	43 (9.07)	237 (70.12)	74 (7.92)	0.330
Very low birth weight (<1500 g), no. (%)	143 (7.55)	31 (21.23)	—	96 (28.40)	—	0.080
Infant in NICU care, yes, no. (%)	490 (25.87)	98 (67.12)	66 (13.92)	196 (57.99)	130 (13.92)	0.701
Infant's delivery status, no. (%)^¶^						**0.034**
Alive	1804 (95.25)	600 (96.77)		1203 (94.58)		
Dead	90 (4.75)	20 (3.23)		69 (5.42)		

P values in the last column are for the comparisons between pre-COVID-19 pandemic (*n* = 620) and pandemic (*n* = 1272) cohorts. Bold value indicates significance at 0.05 level. We did not detect any significance difference between pre-pandemic and pandemic cohorts for preterm and term groups. ^a^, ^b^, ^c^, and ^d^ indicate pre-pandemic cohort with preterm birth, pre-pandemic cohort with term birth, pandemic cohort with preterm birth, and pandemic cohort with term birth, respectively. ^§^The vaginal delivery includes induction. ^¶^Groups were combined to avoid presenting small numbers (<10). No significant association was detected in subgroup comparisons.

**Table 3:** Logistic regression.

	Univariate logistic regression			Multivariate logistic regression		
	Estimates (std. error)	Odds ratio (95% CI)	*P* value	Estimates (std. error)	Odds ratio (95% CI)	*P* value
Pandemic cohort	−0.27 (0.13)	0.58 (0.35, 0.97)	0.036	−0.23 (0.42)	0.56 (0.25, 1.20)	0.595
Infant's gestational age, weeks	0.43 (0.03)	1.53 (1.44, 1.63)	<0.001	0.48 (0.07)	1.39 (1.27, 1.54)	**<0.001**
Very low birth weight (<1500 g)	−2.00 (0.21)	0.02 (0.01, 0.04)	<0.001	−2.06 (0.54)	0.25 (0.08, 0.75)	**<0.001**

Bold value indicates significance at 0.05 level in the multivariate logistic regression model.

## Data Availability

Data may be available upon request and through a DUA overseen by the IRB at the University of Mississippi Medical Center. JReneker@UMC.edu may be contacted for such inquiries.

## References

[1] Birth Table (2018). *Mississippi State Department of Health Office of Vital Records and Statistics. Mississippi Statistically Automated Health Resource System*.

[2] Mississippi State Department of Health (2019). *Mississippi Maternal Mortality Report 2013-2016*.

[3] Mississippi State Department of Health (2020). *Infant Mortality Report 2019*.

[4] Monaghesh E., Hajizadeh A. (2020). The role of telehealth during COVID-19 outbreak: a systematic review based on current evidence. *BMC Public Health*.

[5] Demeke H. B., Merali S., Marks S. (2021). Trends in use of telehealth among health centers during the COVID-19 pandemic—United States, June 26-November 6, 2020. *MMWR. Morbidity and Mortality Weekly Report*.

[6] Behrman R. E., Butler A. S., Institute of Medicine Committee on Understanding Premature Birth and Assuring Healthy Outcomes (2007). The National Academies Collection: Reports funded by National Institutes of Health. *Preterm Birth: Causes, Consequences, and Prevention*.

[7] DeNicola N., Grossman D., Marko K. (2020). Telehealth interventions to improve obstetric and gynecologic health outcomes. *Obstetrics and Gynecology*.

[8] American College of Obstetricians and Gynecologists (2020). Implementing telehealth in practice. *Obstetrics and Gynecology*.

[9] Madden N., Emeruwa U. N., Friedman A. M. (2020). Telehealth uptake into prenatal care and provider attitudes during the COVID-19 pandemic in New York city: a quantitative and qualitative analysis. *American Journal of Perinatology*.

[10] Aziz A., Zork N., Aubey J. J. (2020). Telehealth for high-risk pregnancies in the setting of the COVID-19 pandemic. *American Journal of Perinatology*.

[11] American College of Obstetricians and Gynecologists (2021). Antepartum fetal surveillance: ACOG practice bulletin, number 229. *Obstetrics & Gynecology*.

[12] Lassi Z. S., Ana A., Das J. K. (2021). A systematic review and meta-analysis of data on pregnant women with confirmed COVID-19: clinical presentation, and pregnancy and perinatal outcomes based on COVID-19 severity. *Journal of Global Health*.

[13] Timircan M., Bratosin F., Vidican I. (2021). Exploring pregnancy outcomes associated with SARS-CoV-2 infection. *Medicina*.

[14] Morris R. J., Owens A., Bofill M. (2020). COVID-19 and pregnancy: the UMMC experience - 50 cases and counting. *Journal of the Mississippi State Medical Association*.

[15] Camm A. J., Fox K. A. A. (2018). Strengths and weaknesses of ’real-world’ studies involving non-vitamin K antagonist oral anticoagulants. *Open Heart*.

